# Health benefits of serious involvement in leisure activities among older Korean adults

**DOI:** 10.3402/qhw.v9.24616

**Published:** 2014-07-23

**Authors:** Junhyoung Kim, Naoko Yamada, Jinmoo Heo, Areum Han

**Affiliations:** 1 Department of Recreation, Parks, and Leisure Services Administration, Central Michigan University, Mt Pleasant, MI, USA; 2 Division of Sport Science, Pusan National University, Pusan, South Korea; 3 Department of Recreation, Park and Tourism Sciences, Texas A & M University, College Station, TX, USA

**Keywords:** Serious leisure, health, well-being, older Korean adults

## Abstract

The existing literature suggests that serious engagement in leisure activities leads to happiness, life satisfaction, and successful aging among older adults. This qualitative study was used to examine the benefits of serious involvement in leisure activities among older Korean adults who were members of a sports club. Using an analytic data analysis, we identified three main themes associated with the benefits of serious engagement in leisure activities: 1) the experience of psychological benefits, 2) the creation of social support, and 3) the enhancement of physical health. These themes indicate that, through serious involvement in certain physical activities, participants gain various health benefits, which may contribute to successful aging.

Leisure activities have been advocated as a means by which to improve the health and well-being of older adults (McAuley & Rudolph, 1995). Previous research has provided evidence that participation in leisure activities plays an important role in enhancing the quality of life and life satisfaction among older adults (Gill, Williams, Williams, Butki, & Kim, 1997; McAuley & Rudolph, 1995). Such positive outcomes are closely associated with successful aging (Brown, McGuire, & Voelkl, 2008). The major contribution of leisure involvement to successful aging is that leisure activities offer a context in which older adults can improve their physical functions, enhance positive feelings and emotions, and promote social interactions (Leigey, Irrgang, Francis, Cohen, & Wright, 2009; McCrory, Salacinski, Hunt, & Greenspan, 2009; Orsega-Smith, Payne, & Godbey, 2003). For example, Orsega-Smith et al. (2003) analyzed the physical and psychosocial characteristics of older adults who participated in a community-based exercise program and presented that those who participated more frequently gained better physical, psychological, and social benefits than those who did not participate.

In the context of leisure activities, older adults may actively pursue certain activities with commitment and seriousness. In order to advance their leisure skills and techniques, older adults may invest their time and effort in these activities and share their experiences with others. A variety of senior sports club organizations and sports events exist to provide and encourage the engagement of older adults in activities. For example, older competitive athletes expressed a strong attachment to their particular activities and gained various benefits from participation, such as the development of friendships and the experiences of youthfulness (Dionigi, 2006). In addition, studies have shown that serious involvement in leisure activities leads to improved health and psychological well-being among older adults (Nets, Wu, Becker, & Tenenbaum, 2005; Poon & Fung, 2008).

In order to understand the context of serious involvement in certain activities, Stebbins (1992, 2001) introduced a term of “serious leisure” that illustrates active leisure engagement with commitment and dedication. He suggested that amateur, hobby, and club activities are examples of serious involvement in leisure activities. In addition, Merrill, Shields, Wood, and Beck (2004) showed that older adults who were dedicated to their engagement in competitive sporting activities obtained psychological benefits, such as joy and positive emotions, from their participation. Brown et al. (2008) conducted a qualitative research study in order to explore the benefits received by older shag dancers from the serious leisure perspective. They found that their participation in shag dancing facilitated lifelong learning, fostered personal growth, enhanced their sense of happiness and enjoyment, and improved their life satisfaction.

Misener, Doherty, and Hamm-Kerwin (2010) studied the serious leisure experiences of older adults involved in a community organization, such as volunteering activity. These authors showed that, through serious involvement in volunteering activity, older adults developed the ability to adapt to new life circumstances (e.g., retirement) and increased their life satisfaction and satisfying leisure experiences. Such benefits served as important vehicles for facilitating successful aging (Misener et al., 2010). In addition, serious engagement in leisure offers an opportunity for older athletes to create a social and emotional support system (Costanzo & Walker, 2008; Sasidharan, Payne, Orsega-Smith, & Godbey, 2006; Shaw & Janevic, 2004). These studies demonstrated that, as a result of participation in such activities, older athletes increased their positive social interactions and expanded their social networks.

In spite of the benefits of serious involvement in leisure activities, the majority of prior studies have mainly focused on older adults in Western cultures (Brown et al., 2008; Heo, Culp, Yamada, & Won, 2013; Heo & Lee, 2010). In addition, few studies have investigated the health-related benefits of serious involvement in activities in the context of Eastern cultures. In this article, we sought to explore the benefits of serious engagement in leisure activities among older Korean adults who participated in a sports club. This study may provide insights into the health benefits of serious involvement in sports clubs among older adults from Eastern cultures.

## Literature review

### Serious involvement in physical leisure activities

A positive relationship exists among older adults between leisure activity engagement and health-related outcomes (Buettner & Fitzsimmons, 2002; Plonczynski, 2000). Prior research has provided evidence that leisure activity involvement is associated with a reduced risk of disease and chronic illnesses (Grove & Spier, 1999), improved physical strength and functions (Skelton, Young, Greig, & Malbut, 1995), and increased muscle strength and joint flexibility (Hellman, 1997; Plonczynski, 2000). In addition, researchers have found that through leisure activity, older adults reported that they experienced positive psychological and mental health aspects, such as happiness, enjoyment, and positive emotions and feelings (Buettner & Fitzsimmons, 2002; Heo & Lee, 2010).

Substantial research has suggested that older adults who actively express an attachment to certain activities have a strong desire to maintain their involvement and create social and emotional connections with other participants who have similar interests (Siegenthaler & O'Dell, 2003; Stebbins, 2001). For example, Benjamin, Edwards and Bharti (2005) analyzed predictors of leisure activity intentions and behaviors of older adults and found that older adults involved in activities in a serious manner were likely to have a stronger intention to maintain their involvement than those not involved in a serious manner.

Siegenthaler and O'Dell (2003) conducted in-depth interviews with older golfers in order to assess their serious involvement in the sport and explored the relationship between serious golf experiences and successful aging. They identified several personal and social benefits (e.g., social interactions, enjoyment, and physical benefits) associated with the participants’ high level of involvement in golf. In their study, the older adults who reported a higher level of seriousness had a better sense of awareness of their activity benefits than others in the study. As a result of their serious golf experiences, these older adults enhanced their perception of successful aging.

A qualitative study conducted by Major (2001) explored the benefits of serious involvement in activities among runners. He identified three major categories of benefits: sense of accomplishment (i.e., self-confidence, power, and control), health and fitness (i.e., physical benefits and stress relief), and social affiliation. He also addressed the importance of examining the benefits of serious experiences in activities. This study indicates that serious involvement in activities may play an important role in improving the health and well-being of participants.

In addition, Brown et al. (2008) attempted to understand the experience of shag dance among older adults who actively pursued it. They concluded that older adults who pursued shag dance in a serious manner gained various benefits related to successful aging, such as the development of new skills, personal growth, life satisfaction, interpersonal relationships, enjoyment, and happiness. They also suggested that superficial involvement in shag dance was not associated with the identified outcomes (e.g., personal growth, contentment, relationships, and satisfaction) among older adults.

## Methods

This study was designed to capture the benefits of serious involvement in leisure activities among older Korean adults. In this article, we focused on older Korean adults who participated in a sports club in the context of serious leisure. Using the constant comparative method (Merriam, 1998), we simultaneously and continuously analyzed the data and created the categories.

### Participants

The criteria for this study were that participants had to be aged over 65 years, been members of a sports club over years, and have the ability to read and understand Korean.

A total of 10 participants voluntarily participated in this study. On average, participants have been members of sports club more than 15 years. Most of them sought to maintain their leisure activities before retirement. The range of their ages was from 66–83 years (Mean=71). Three of the participants were male, and seven were female. All of the participants were retired and had a bachelor's degree. On the basis of theoretical saturation, as suggested by Mack, Woodsong, MacQueen, Guest, and Namey (2005), we reached a point where new ideas and/or relationships did not emerge from the data when we interviewed the ninth participant. In order to ascertain the saturation point, we conducted another interview and then studied the data. Each investigator agreed that the saturation point had been reached. The participants were compensated with approximately $20 USD.

### Data procedure and collection

We approached program directors of community-based physical activity clubs and organizations for older adults in a metropolitan area of South Korea. With the directors’ permission, we placed a research flyer containing the study information, including a brief description of the study and our contact information, at the club. Within a few weeks, several potential participants had contacted us. Upon contact, we provided them with detailed information about the study (e.g., confidentiality, participants’ rights, and interview procedures). In order to make sure that the participants did not have any intellectual challenges, such as dementia, which would limit their abilities to participate in the in-depth interviews, we evaluated each potential participant's ability to read and understand Korean with some general questions about South Korea. The university institutional review board approved these procedures.

In-depth interviews were used to collect the data. The interviewees chose the place and time that were most convenient for them for the interviews. Each interview lasted between 50 and 90 min. We developed semi-structured interview questions by incorporating the interview strategy suggested by Spradley (1979) into our study. The questions consisted of grand and mini tour questions so that we could capture the benefits of the participants’ engagement in activities. Each interview began with grand tour questions, such as “When did you become a member of this activity club?” and “Tell me about your general experience being a member of this club.”

The interviews became more structured and focused on the benefits of the participants’ involvement in leisure activities during the mini tour questions. Examples of these questions were “Why did you participate in this activity?” “What benefits do you experience when you participate in this activity?,” and “Did you have any challenges in regard to participating in this activity?” This interview strategy enabled us to capture the benefits that the participants gained through their sports club activities.

### Data analysis and trustworthiness

We used an analytic data analysis as this approach has helped researchers extract solid and accurate relationships from data (Gerson & Horowitz, 2002) in earlier studies. In addition, this analytic data analysis method increased the accuracy of the data patterns (Gerson & Horowitz, 2002). We followed a three-step data analysis: 1) the creation of the open coding, 2) the generation of axial coding, and 3) selective coding through an interconnected storyline.

After we created the raw data, we executed a line-by-line level of analysis so that we could create the open category and describe the properties of each category (Charmaz, 2006; Gerson & Horowitz, 2002). Each investigator followed this process of data analysis individually and shared their open category with the other researchers. Because we had several opening codes, we used the constant comparative method (Merriam, 1998) in order to compare and understand these categories. By comparing each opening category, we generated implicit meanings for each of the emerging categories. After reviewing the opening categories, we identified the relationships among the categories and created subcategories for each opening category. In the final stage of the data analysis, we produced the core themes and subthemes, including rich quotes and narrative descriptions that were extracted and interpreted from the axial coding.

We sought to strengthen the credibility of the data using several strategies. First, we used a back-translation process in order to increase the accuracy of the translations from Korean to English. We invited another investigator who was not on the research team to verify the translations. We provided him with random paragraphs from the data and, via a conference call, evaluated the quality of the translations. No significant discrepancies in the translations for the selected paragraphs were found. In addition, each investigator had rich qualitative research experience and participated in analyzing and coding the transcripts as well as had ongoing discussions with regard to the data analysis. The last technique we used was a member-checking process suggested by Lincoln and Guba (1985). We provided a brief description of the identified themes and their interpretations to the participants. They expressed satisfaction of our analysis and interpretations.

## Results

We identified three main themes within the benefits of leisure activities: 1) the experience of psychological benefits, 2) creation of social support, and 3) enhancement of physical health. These themes indicate that, through serious involvement in certain physical activities, participants gain various health benefits, which may contribute to successful aging.

### The experience of psychological benefits

The experience of psychological benefits was one of the most salient themes identified from the data. All of the participants mentioned that they gained various psychological benefits. They mentioned that such psychological benefits served as important motivation that drove their participation in the activities in a serious manner. Two subthemes emerged from the data related to psychological benefits: 1) enjoyment and positive feelings, and 2) enhanced self-esteem and confidence.

#### Enjoyment and positive feelings

The participants used several expressions to describe how their participation in the sports club brought them joy and enjoyment, including “I always feel happy when I am involved with dance,” “practicing with my teammates has made me so happy,” and “competing with others gives me energy and [provides me with] enjoyment.” They made significant accomplishments through their senior sports tournaments, which allowed them to enjoy their participation and increase their happiness.

Even though some of the participants faced numerous challenges, such as physical challenges related to age, they said that they would not consider stopping their participation in their activities. As a result, they developed a sense of perseverance and gained positive feelings and emotions. For example, one participant, who was involved with dance, said:

Sometimes, I encountered challenges, physically and psychologically, when I practiced with others, but there is always a feeling of joy. I believe that I developed a strong mentality through it (dance) and it is so much fun especially when we feel that we could achieve something. Regardless of how old we were, we were so happy to show others that we could still do this activity.

#### Enhanced self-esteem and confidence

Some of the participants believed that they improved their self-esteem and confidence through their activities because they acquired advanced skills and techniques. For example, one gateball, a mallet team sports similar to croquet, participant said that he tended to demonstrate his gateball skills to others and, with his advanced skills, saw his confidence increase. In addition, in various gateball tournaments, he received trophies and tournament awards, which also caused his self-esteem and confidence to become enhanced.

Another participant, who was a member of the aerobics club, said,

I was a leader on a team and helped my teammates improve their skills and techniques. We practiced together and encouraged each other …. In the end, we received some awards from some competitions and I just felt like everything had paid off. As a leader, I also developed the confidence necessary to undertake challenges with my teammates.

She also said that she was proud of her accomplishments.

Some of the participants thought that, by participating in sports club activities, they were able to relieve their stress and cope well with challenges. For example, they mentioned that, after practices, they shared their personal life experiences and created an emotional and social support network for each other. These emotional connections helped them to relieve their stress. In addition, they stated that active involvement in their activities helped to decrease their overall levels of tension and elevate and stabilize their moods. According to one participant, “Doing this (dance) makes me laugh and removed my stress. Sometimes, when I lay down at night, I think of those moves.” Replaying her dance moves in her head, while exhausted, allowed her to reduce her negative feelings and elevate her mood.

Based on the participants’ personal experiences, serious involvement in leisure activities has offered opportunities for older Korean adults to gain psychological benefits, such as positive feelings and emotions, and has enhanced self-esteem, confidence, and coping strategies. Even though they encountered some challenges associated with their age, they developed the ability to overcome these challenges and actively pursued their participation in their chosen activities, which contributed to their psychological benefits.

### The creation of social support

The creation of social support was identified as another salient theme that emerged from the data. By participating in various sports club activities, most of the participants mentioned that they created and fostered positive social interactions with other participants and developed intimate friendships. Some of the participants described themselves as extremely enthusiastic participants. Thus, they tended to share their interests and experiences related to the activities and easily provided support for one another. For example, one participant mentioned that the club members shared common goals and interests and treated each other like family members. Similarly, another participant stated:

I really enjoyed practicing with my team members and as it provided us with opportunities to get to know each other well. After practices, we hung out, went to eat, and traveled together when we went to competitions. We have become good friends, which helps the time go faster.

He also believed that his team members provided him with good advice when he needed it on personal matters.

According to most of the participants, their sports club activities offered them opportunities through which to establish a sense of friendship with other participants. They stated that their friendships were intimate and well-established using similar expressions, such as “I made close friendships,” “we became really good friends,” and “our friendships are really deep.” One participant shared her life experiences in order to illustrate how much she valued her friendships with the other club members.

We really valued our friendships and this is the most important quality in my life. Our friendships became much stronger and, when we went to competitions, we supported each other regardless of the tournament results. Also, we always learned from each other.

Some of the participants thought that their family members became significant supporters of their activity participation. They felt that, sometimes, their family members sacrificed their time and energy in order to allow them to practice and/or go to competitions. Based on the strong support and encouragement from family members that they received, the participants mentioned that their family relationships had gotten stronger and were built more on trust and affection than ever before. According to one participant,

My children really love it when I go to competitions. They say thing like “Mom, did you really go to that competition?” and “If someone taped the event, we need to watch it.” They actually called people who taped the event and, somehow, downloaded the videos to their computers and then told me, “Mom, you are so cool!” They really love it. I was a little embarrassed the first time, but, since my children like what I do, I think I not as shy about it as I used to be. People around me tell me that I've changed. I used to have hard time looking into others’ eyes, but I'm not like that anymore.

She also felt that her relationships with her children and grandchildren were greatly improved because of her participation in these activities.

These examples have shown how the participants’ leisure activities have provided them with rich opportunities to create and foster social and psychological support systems. The participants received various emotional and social supports from their family and club members. It seems that serious involvement in activities plays an important role in expanding participants’ social networks and increasing their positive social interactions.

### The enhancement of physical health

We identified the enhancement of physical health as another salient theme in our study. Some of the participants experienced positive changes with regard to several physical components as a result of their involvement in their activities. They said that they gained physical strength and endurance due to the practice sessions. One participant who played gateball said that he increased the number of games that he played because of his enhanced physical strength. He also felt that he had improved his quality of sleep and overall physical health. Another gateball participant said,

When I play gateball, I usually play about five games during the day. You may end up walking about 500 steps. You also have to think a lot. It's not just hitting balls. You need to have wisdom and strategies to win. Since you think a lot, I guess it can prevent Alzheimer's disease. I saw that information in a newspaper.

He also believed that he improved his flexibility and the ability to react quicker due to his participation.

Some of the participants who had illnesses thought that their health had significantly improved because of their participation. One participant who had colorectal cancer said that her health was getting better because of her participation in gateball. Another participant stated,

I am a Vietnam War veteran and I got injured pretty badly during the war. Since then, I've had pain in my back and neck. I have lived with the pain for more than 30 years and have done lots of things, like alternative therapies and worked with therapists from different disciplines; however, it never got better. One day, I walked by a downtown square and saw people practicing aerobics. The music was quite enjoyable. That day, I thought to myself that I might be able to change my physical constitution and, hopefully, get better if I practiced something like that. That's how I got started and it actually helped me a lot. Of course, it's fun and I've gained some endurance in my body and lost some of the pain.

He gained various physical benefits through aerobic exercises, such as a reduction in muscle pain and increase in endurance.

Another interesting example came from a participant who got her husband to exercise with her.

My husband did not like exercising and was reluctant to do it. When I asked him to go with me, he would refuse and tell me, “I'm weak and I don't wanna waste my energy.” One of his kidneys did not work correctly. However, not that I've gotten him to exercise with me, he can't live without it. He exercises at home and then again for an hour or two at the senior center. Now, I have to tell him not to overwork him. He had a physical exam recently and was told that he is very healthy.

A few of the female participants said that they lost some weight after they joined the sports club. Their continuous involvement in activities also helped them to facilitate healthy behaviors, such as eating healthily and exercising more often. Another comment from one of the female participants was that she improved her balance and posture through jazz and other types of dance. She said that many older adults have poor posture, but, through dance, she was able to greatly enhance her muscle strength, in particular in her legs, and other muscles that affect her posture. She said that, even though she occasionally experienced muscle pain while dancing, she would not stop her activities.

Based on these statements, we learned that the participants gained various physical benefits, such as strength, flexibility, agility, balance, and a good posture, from their participation. We also learned that their participation helped them to develop the ability to overcome negative challenges.

## Discussion

This qualitative study examined the benefits of sports club activities in the context of serious leisure among older Korean adults. The results showed that serious involvement in sports club activities provided the participants with health-related benefits, such as psychological, social, and physical benefits. The findings of this study indicate that leisure activities produce opportunities for older Korean adults to increase their positive psychological feelings, enhance positive social relationships, and improve their physical functions. Such positive outcomes of leisure activity involvement may be associated with successful aging.

The body of leisure literature demonstrates the value of leisure activities for physical, psychological, and social health benefits (Mannell, 2007; Paganini-Hill, Kawas, & Corrada, 2011). The findings of this study are consistent with past leisure literature. This study suggests that psychological benefits are the main outcomes that older Korean adults said they obtained. This result holds true because older adults may experience depressive symptoms, isolation, and anxiety related to their adaptation to a new society (Mio, Barker-Hackett, & Tumambing, 2006; Shim & Schwartz, 2007). This study also suggests that serious involvement in activities helped older Korean adults to cope with negative feelings and gain increased happiness and positive feelings. Furthermore, it seems that leisure activity involvement plays an important role in improving the psychological well-being of older adults.

Considerable research has stressed the importance of serious involvement in activities as it increases the quality of life and successful aging of older adults (Brown et al., 2008; Heo, Lee, Pedersen, & McCormick, 2010). Unfortunately, prior studies have mainly been focused on the benefits of serious leisure involvement among predominantly White, middle-class, and non-Hispanic older adults. This study extends the scope of serious leisure benefits to the Eastern context in which older Korean adults gained various health-related benefits from their participation. It seems that, regardless of the cultural settings, older adults can improve their health and well-being through leisure activities.

Stebbins (2001) suggested that serious involvement in leisure activities produces opportunities for the participants to gain personal and social benefits. The researchers have identified personal benefits as an outcome of serious involvement in leisure activities, such as an increase in confidence and self-esteem, personal achievements, and an acquisition of advanced skills (Baldwin & Norris, 1999; Mackellar, 2009; Major, 2001; Patterson, 2000; Shipway & Jones, 2007). The social benefits that manifested themselves included positive social interactions and social relationships, intimate friendships, and expanded social networks. This study adds to the current body of knowledge that shows that serious involvement in leisure activities increases the psychological, physical, and social well-being of older adults. In addition, this study extends prior studies by supporting the contention that serious involvement in leisure activities leads to health benefits, such as life satisfaction and happiness (Heo et al., 2013; Kim et al., 2012).

The participants in this study demonstrated these qualities of serious involvement in activities. For example, even though the older Korean participants encountered challenges associated with age, they were willing to pursue leisure activity participation, which helped them to develop a sense of perseverance. In order to advance their skills and techniques as they are associated with their particular activities, the participants exerted significant effort and competed with others to demonstrate their advanced skills. In addition, they created their own identities as activity enthusiasts and shared their personal experiences related to activity participation, which created a unique world for them. These six characteristics may be associated with health benefits of older Korean adults.

In terms of the pursuit of happiness, Suh and Oishi (2004) stated, “happiness in North America is essentially attained via personal achievement and self-esteem, whereas happiness in East Asia is attained via supportive social relationships” (p. 220). The findings of this study support this idea that social support from others through leisure activities may be an important element for enhancing psychological well-being and health among Korean older adults. In addition, Korean culture emphasized the value of in-group decision and collectivistic ideas (Uchida, Norasakkunkit, & Kitayama, 2004) and when Korean older adults determined their leisure activity engagement, such cultural characteristics may influence their decision making. In order to provide the effective activity service, the cultural aspects of leisure participation must be understood from an older adult's perspective.

There are some limitations that we need to address in the study. First, we approached the leisure behaviors of older Korean adults from the serious leisure perspective. In this study, we did not actually measure the degree of the participants’ serious leisure qualities and their health-related benefits. If future studies examined the relationship between serious leisure involvement and its health benefits, then it may provide rich data information and insightful ideas on how serious involvement can lead to health improvements.

Another limitation exists regarding the nature of the participants’ characteristics. We recruited older Korean adults who were currently physically active and participated in various sports club activities. This study may provide limited information on the general population of older adults because many older adults may have a fear of physical activity participation or be reluctant to engage in them because of their ages. It would be beneficial for researchers to expand the scope to the older adult population in general and explore what motivates or prevents older adults from participating in the activities.

The final limitation of this study was that it does not determine which factors motivate older adults’ serious engagement in activities. The participants in our study maintained leisure interests and desire in order to pursue their activity participation. Several factors may influence their active engagement, such as past experiences, health status, and social support. It may be helpful for future research to explore what factors influence older adults’ participation in activities.
